# Role of the ABL tyrosine kinases in the epithelial–mesenchymal transition and the metastatic cascade

**DOI:** 10.1186/s12964-021-00739-6

**Published:** 2021-05-22

**Authors:** Jillian Hattaway Luttman, Ashley Colemon, Benjamin Mayro, Ann Marie Pendergast

**Affiliations:** Department of Pharmacology and Cancer Biology, Duke University School of Medicine, 308 Research Drive, C-233A LSRC Bldg., P.O. Box 3813, Durham, NC 27710 USA

**Keywords:** ABL kinases, Signaling, EMT, Metastasis

## Abstract

The ABL kinases, ABL1 and ABL2, promote tumor progression and metastasis in various solid tumors. Recent reports have shown that ABL kinases have increased expression and/or activity in solid tumors and that ABL inactivation impairs metastasis. The therapeutic effects of ABL inactivation are due in part to ABL-dependent regulation of diverse cellular processes related to the epithelial to mesenchymal transition and subsequent steps in the metastatic cascade. ABL kinases target multiple signaling pathways required for promoting one or more steps in the metastatic cascade. These findings highlight the potential utility of specific ABL kinase inhibitors as a novel treatment paradigm for patients with advanced metastatic disease.

**Video abstract**

**Supplementary Information:**

The online version contains supplementary material available at 10.1186/s12964-021-00739-6.

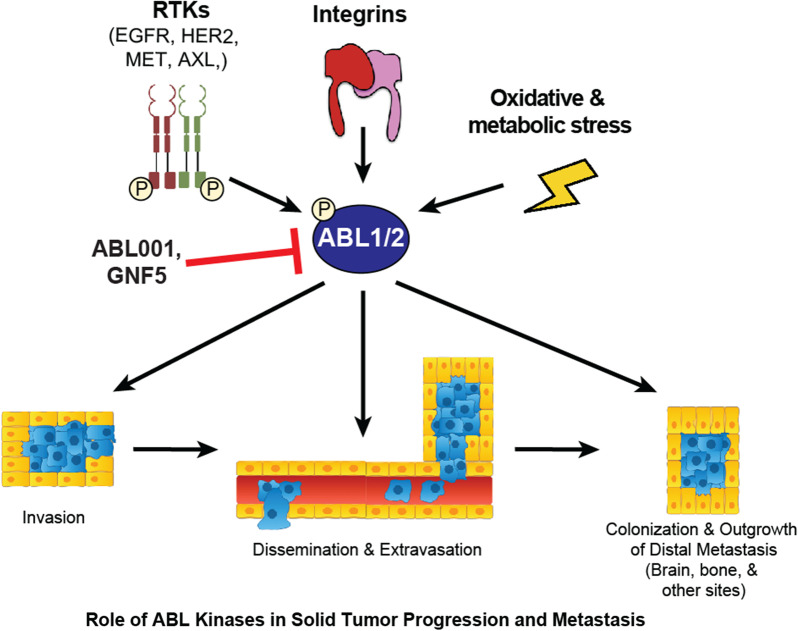

## Background

Tyrosine kinases regulate a vast array of cellular signaling networks necessary for processes such as survival, growth, migration, and invasion. Regulation of these processes is required for proper mammalian development and cellular homeostasis [1]. The Abelson (ABL) family of tyrosine kinases ABL1 (c-ABL) and ABL2 (ABL-related gene, ARG) regulate diverse cellular processes during development and normal homeostasis, but ABL kinases are aberrantly activated during tumor progression, metastasis, tissue injury responses, inflammation, neural degeneration and other diseases [2–11]. ABL kinases are activated by diverse stimuli including but not limited to growth factors, adhesion receptors, chemokines, oxidative stress, and DNA damage [12]. Upon activation, ABL kinases can alter the cytoskeletal network necessary for cell migration, adhesion, polarity, phagocytosis and motility [13]. In solid tumors, activated ABL kinases can promote invadopodia formation, invasion, and diverse cellular processes implicated in the epithelial–mesenchymal transition (EMT) and subsequent steps in the metastatic cascade.

ABL1 was initially identified as a driver of leukemia in mice and humans [14, 15]. Subsequently, ABL1 and ABL2 were shown to promote solid tumor progression and metastatic dissemination [4, 6, 11, 16–18]. In the context of solid tumors, ABL kinases are upregulated due to enhanced gene expression and/or enzymatic activation by oncogenic drivers, such as receptor tyrosine kinases (RTKs) and chemokine receptors [12]. Upon activation, ABL kinases can potentiate cancer cell survival, proliferation, migration, and invasion, depending on the cellular context. In this review, we will focus on the role of ABL kinases in regulating downstream targets implicated in EMT as well as distinct cellular processes required for metastatic dissemination. Recent reports have revealed that inhibition of the ABL kinases can decrease tumor outgrowth and impair metastatic spread, indicating the potential use of ABL kinase inhibitors for the treatment of some solid tumors with activated ABL kinases [2, 4–6, 10, 11, 17, 19–22].

## ABL kinases regulate EMT-related cellular processes

The EMT program is characterized by the loss of epithelial characteristics and acquisition of mesenchymal traits [23]. The EMT program is dependent on activation of a transcriptional program that includes a panel of transcription factors such as SNAIL, SLUG, ZEB1, and TWIST [24]. These and other factors are activated downstream of diverse signaling pathways initiated by RTKs, chemokine receptors and adhesion receptors, which in turn activate protein kinases such as the ABL kinases and co-transcriptional regulators that converge on one or more EMT transcription factors. EMT has been associated with enhanced tumor invasion, migration, and metastasis, as well as increased cancer cell stemness and chemo-resistance [23]. A hallmark of EMT is the loss of epithelial polarity and dissolution of cell–cell junctions by decreasing expression of adhesion receptors, such as E-cadherin, or disrupting the localization of β-catenin and other E-cadherin-associated proteins at sites of intercellular adhesion, resulting in enhanced cell migration and invasion [24]. Accumulating evidence support a role for ABL kinases in the regulation of cell–cell adhesion, migration and invasion, which are processes implicated in EMT [25]. Active ABL kinases facilitate changes in actin dynamics and promote remodeling of adherens junctions, which is necessary for EMT [26, 27]. Moreover, ABL kinases activate and promote nuclear accumulation of the TAZ transcriptional co-activator in breast and lung cancer cells, and TAZ has been shown to promote EMT [4, 6, 11, 28]. ABL kinases have also been shown to regulate expression of the EMT transcription factors ZEB1, TWIST1, SNAIL1, and/or SLUG in a cell context-dependent manner [10, 20, 29]. Here we review the unique properties of the ABL kinases and their role in the regulation of multiple cellular processes implicated in the EMT program.

### Structural domains and regulation of ABL kinases

**Fig. 1:**
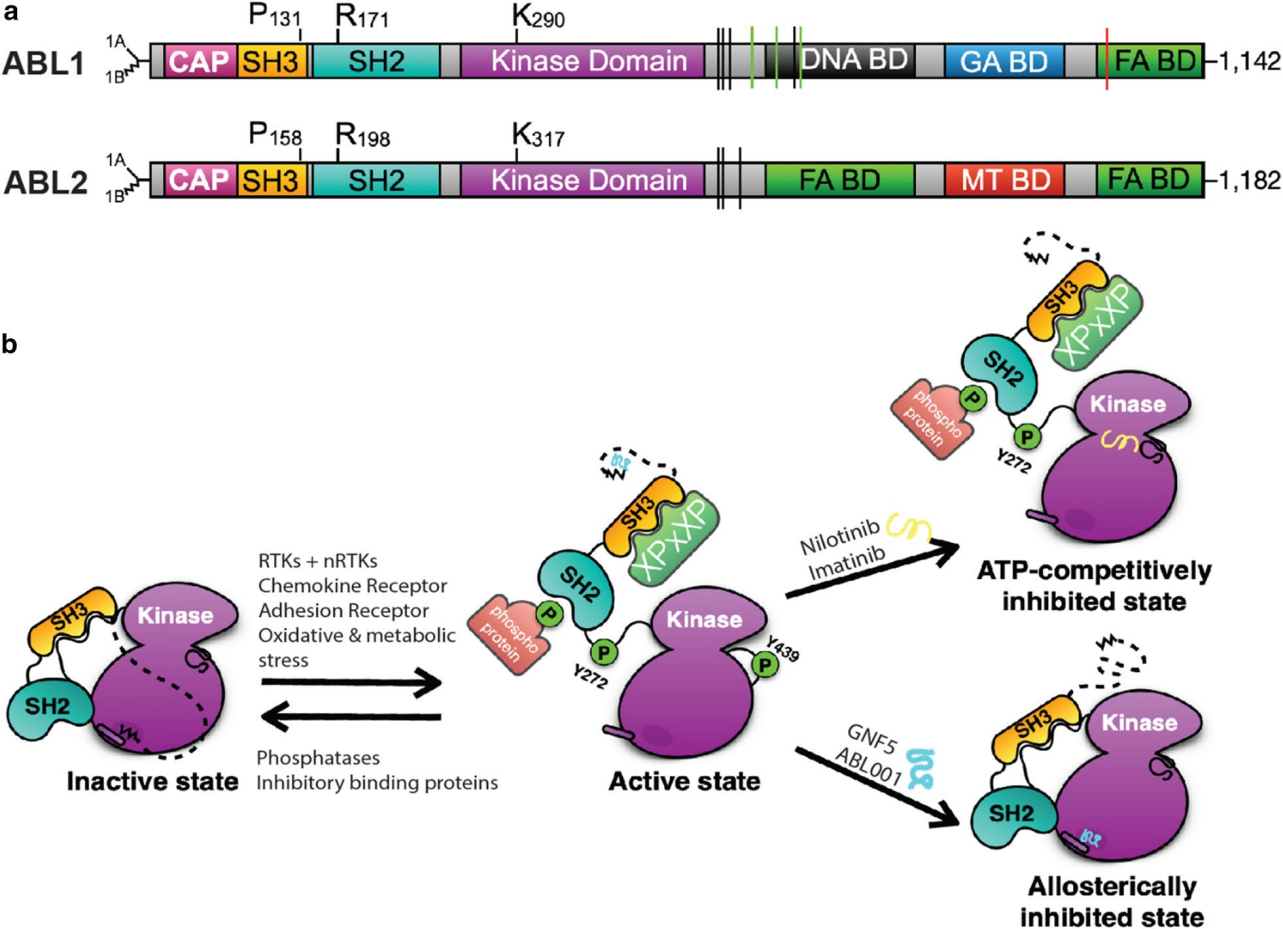
Representation of the ABL structural domains and regulation of ABL kinase activity. **a** There are 2 major splice variants of *ABL1* and *ABL2*, the 1A isoforms (straight line) and the 1B isoforms (jagged line); numbering uses the 1B isoform. The amino (N)-termini of the ABL kinases contain the SRC homology 3 (SH3), SH2 and SH1 (tyrosine kinase) domains. The carboxyl C-termini of the ABL kinases are divergent with only a conserved filamentous (F)-actin-binding domain (BD) between both paralogs. ABL1 has a globular (G)-actin-binding domain and a DNA-binding domain, whereas ABL2 has a second internal F-actin-binding domain and a microtubule (MT)-binding domain. ABL1 has three nuclear localization signal (NLS) motifs (three green lines located near the SH1 domain) and one nuclear export signal (NES) (single red line in FA BD) in its C terminus. Both paralogs have conserved XPxXP motifs to mediate protein–protein interactions (denoted as black vertical lines in both structures). P131/158L is a mutation that destroys SH3-mediated interactions and R171/198 K is a mutation that destroys SH2-mediated interactions. L290/317R are kinase inactivating mutations. **b** Inactive and active forms of the ABL kinases are regulated by dynamic intramolecular interactions that modulate ABL kinase activity. The SH3 domain binds to the linker sequence connecting the SH2 and the kinase (SH1) domains, and the SH2 domain interacts with the C-terminal lobe of the kinase domain forming an SH3–SH2 clamp structure locking the kinase in an inactive state. The dashed line represents ABL N-terminal sequences upstream of the SH3 domain that fold over and bind to the myristoyl group in a pocket of the C-lobe of the kinase domain. The myristoylated residue is present in in the N terminus of the ABL 1B isoforms and creates a hydrophobic pocket within the C-lobe of the kinase domain that stabilizes the auto-inhibited conformation. Activation of the ABL kinases by diverse stimuli disrupts the inhibitory intra-molecular interactions. Phosphorylation within the activation loop of (Y412 in ABL1; Y439 in ABL2) as well as within the SH2-kinase domain linker (Y245 in ABL1; Y272 in ABL2) stabilizes the active conformation. Binding of pharmacological inhibitors to the ATP-binding site (Nilotinib or Imatinib) or to the allosteric site (GNF5 or ABL001) disrupts these interactions and causes kinase inhibition by eliciting different conformations

ABL kinases are a family of non-receptor tyrosine kinases (nRTKs) consisting of two paralogs, ABL1 and ABL2 (Fig. 1a). ABL1 was first discovered as the cellular homolog of the Abelson murine lympho-sarcoma virus (A-MuLV) [14]. It was later discovered that constitutive activation of ABL1 upon fusion with the breakpoint cluster region (BCR) generated the BCR-ABL1 oncoprotein responsible for driving several forms of human leukemia [15]. ABL2 was later identified using a sequence homology search [30]. While the oncogenic ABL proteins exhibit constitutively active kinase activity, the endogenous ABL kinases cycle between inactive and active conformations dependent on intra-and inter-molecular interactions (Fig. 1b).

The amino (N)-terminal domain of the ABL kinases contains highly conserved regulatory SRC homology (SH) 3 (SH3) and SH2 domains, followed by the kinase or SH1 domain. While the SH3-SH2-SH1 cassette is shared by 19 of the 22 human non-receptor tyrosine kinases (nRTKS), the SH3-SH2-SH1 domains of human ABL1 and ABL2 are more highly conserved (92%) to each other than the corresponding domains of any other nRTKs and their closest respective paralogs or orthologs [31]. SH3 domains canonically bind proline-rich peptides that form left-handed polyproline type II helices [32]. SH3 domains exhibit a wide-array of ligand (PxxP) specificities that have been broadly divided into two major classes: class 1 which bind a (K/R)xxPxxP motif and class 2 which bind a PxxPx(K/R) motif [32]. The consensus binding motif of the ABL SH3 domain is divergent from both class 1 and class 2 with a target sequence of PPx(F/W/Y)xPPP(A/G/I/LV) [33]. Further, recent work revealed that the ABL2 SH3 domain can bind a proline-independent sequence [34].

SH2 domains canonically bind tyrosine phosphorylated peptides. The ABL SH2 and SH1 have co-evolved to exhibit a similar consensus binding motif (VYxxP) [31]. The SH3 and SH2 domains are preceded by an amino (N)-terminal CAP region and together these sequences engage in intra and intermolecular interactions that modulate tyrosine kinase activity. There are two major splice variants of the ABL kinases with alternative start sites (1A- short isoform, 1B- long isoform; numbering in this review uses 1B) [35]. A glycine residue exists in the 1B isoform that becomes myristoylated, and the myristoyl moiety binds to a pocket in the C-lobe of the kinase domain to stabilize the inactive kinase conformation [36]. Activation of ABL kinases by diverse stimuli leads to disruption of intramolecular interactions and phosphorylation of downstream targets (Fig. 1b).

The carboxy (C)-terminal domain of the ABL kinases is encoded by a single exon (Fig. 1a). While the N-terminal CAP-SH3-SH2-SH1 domains are highly conserved (90%), the C-terminal domains are divergent (29%), suggesting potential unique functions of ABL1 and ABL2 [30]. Both kinases have three conserved class 2 PxxP located adjacent to the SH1 domain which mediate binding to proteins containing SH3-binding domains [37]. The ABL1 protein localizes to the nucleus and cytoplasm, and encodes three K/R-rich nuclear localization signals (NLS) and a nuclear export signal (NES) allowing its entrance and egress from the nucleus (Fig. 1a). In contrast, ABL2 lacks these domains and is retained in the cytoplasm [38, 39]. ABL1 contains globular (G)-actin and filamentous (F)-actin binding domains, while ABL2 contains two F-actin binding domains and a microtubule-binding domain [40–43]. The presence of these C-terminal sequences endows the ABL kinases with a unique capacity to integrate diverse stimuli to dynamic changes in the actin and microtubule cytoskeletons.

### ABL-mediated regulation of cell–cell junctions

One of the initial steps of EMT is loss of cell–cell junctions causing dissolution of cell adhesion [24]. ABL-mediated regulation of cell adhesion is cell context-dependent as activation of the ABL kinases in some cancers promotes EMT through dissolution of cell–cell junctions, but in noncancerous epithelial tissues the kinases can support cell adhesion. During homeostasis, epithelial cells adhere to one another through specialized adherens junctions that link neighboring cells via cadherin receptors. Cadherins connect to the actin cytoskeleton via α- and β-catenins and are regulated by Rho GTPases which promote remodeling of the cadherin-catenin complex [44]. ABL kinases have been shown to stabilize cadherin-mediated cell–cell adhesion as genetic knockdown or pharmacologic inhibition of ABL1 and ABL2 disrupts N- and E-cadherin based cell–cell contacts in mouse embryonic fibroblasts (MEFs) [26, 27]. In MEFs, a positive feedback loop forms where engagement of the cadherin-catenin complex activates ABL kinase signaling. Upon activation, the ABL kinases initiate signaling through the Crk/CrkL adaptor proteins to activate Rac, a Rho family GTPase, which in turn strengthens cell–cell contacts by promoting formation and maturation of adherens junctions [26, 27]. In contrast to its effect on Rac, ABL2 inhibits the Rho kinase in MEFs. Rho promotes formation of focal adhesions but, following cell attachment, ABL2 is activated and phosphorylates the Rho inhibitor p190RhoGAP (p190) causing subsequent activation of p190 which localizes to the cell periphery and inhibits Rho [45]. Further, ABL kinases promote differential phosphorylation of vinculin at Y822 in response to engagement of cell–cell but not cell–matrix adhesions, and phosphorylation of vinculin by ABL kinases is required for cadherin-mediated force transmission and cell–cell adhesion, due in part to increased recruitment of β-catenin into the cadherin adhesion complex [46]. Thus, ABL activation in response to cadherin-dependent adhesion signals coordinates cell–cell adhesion and contractility in epithelial cells.

In certain cancers, ABL kinases have a converse role where they promote EMT and disrupt cell–cell junctions. In colon cancer, ABL1 is required for platelet-derived growth factor (PDGF)-induced EMT [47]. PDGF stimulation led to loss of cell–cell contacts and cell scattering while causing cells to transition into a mesenchymal-like phenotype. Loss of ABL1 prevented these changes in cell morphology following PDGF stimulation. Following induction by PDGF, ABL1 phosphorylates p68, a RNA helicase, causing β-catenin nuclear translocation leading to disintegration of cell–cell junctions and transition into a mesenchymal phenotype [47]. In the context of metastatic non-small cell lung cancer cells, active ABL kinases promoted β-catenin nuclear accumulation and activation of WNT signaling partly by decreasing β-catenin interaction with the β-TrCP ubiquitin ligase and subsequent protein degradation [4]. Notably, ABL-mediated β-catenin stabilization and activation of downstream signaling networks promoted metastasis of non-small cell lung cancer cells [4].

### ABL-mediated regulation of cell–matrix connections

Detachment from the extracellular matrix (ECM) enhances the epithelial cell transition into a mesenchymal cell type by releasing constraints from the cell matrix and altering intracellular signaling. Integrins are cellular receptors that facilitate cell adhesion by attaching to ECM proteins including fibronectin, collagen, vitronectin, and laminin [48]. In the context of fibroblasts, integrin attachment to fibronectin promotes ABL1 accumulation at sites of focal adhesion. ABL1 can then translocate to the nucleus relaying integrin signaling [49]. The kinase domain of ABL2 directly interacts with the cytoplasmic tail of β1 integrin and phosphorylates Y783 allowing for the ABL2 SH2 domain to engage with Y783 on β1 integrin and subsequent ABL2 activation [50]. This integrin-dependent adhesion pathway drives ABL2-directed cell migration and cell edge dynamics in fibroblasts by enhancing fibroblast attachment to the ECM. When ABL2 is genetically knocked out, ABL2-null fibroblasts have decreased adhesion turnover and detach from the ECM causing increased cell contractility and faster, uncoordinated movement in comparison to their wild-type counterparts [51]. Similar phenotypes were observed following treatment with the ABL kinase ATP-site inhibitor imatinib which impaired membrane protrusions of cells bound to fibronectin [41, 52, 53]. Thus, the ABL kinases are necessary to promote proper ECM attachment in non-transformed cells.

Conversely, ABL kinases disrupt β1-integrin signaling during the transition into a mesenchymal phenotype in cancer cells. Integrin receptors play a role in maintaining cell polarity as epithelial cells orient their basal surface through adhesion of integrin receptors to the extracellular matrix [54]. Apical-basolateral cell polarity is important as it contributes to the acquisition of cell shape and to the directional transport that characterizes epithelial function [55]. Following dissolution of epithelial cell–cell junctions, apical-basal polarity is lost which is a hallmark of EMT [56]. In the context of kidney epithelial cells, active ABL2 disrupted β1-integrin signaling and localization [57]. Prolonged activation of ABL2 disrupted Rac1-mediated assembly of β1-integrin causing perturbed laminin assembly and inverted epithelial cell polarity reminiscent of the early cellular changes following tumor initiation [57]. Disruption of β1-integrin signaling by active ABL2 was shown to be mediated in part by the Rap1 GTPase, and expression of active Rap1 rescued the polarity inversion phenotype induced by active ABL2 in three-dimensional epithelial cyst cultures. Similar signaling changes were noted in prostate cancer cells where ABL kinase activation caused a decrease in Rap1 activation via phosphorylation of the CrkII adaptor and disruption of the CrkII/C3G complex resulting in decreased β1-integrin affinity without altering β1-integrin levels [58]. Taken together, these reports show a context dependent role of ABL kinases in either promoting cell adhesion and attachment to the ECM or impairing these processes when cells begin to undergo EMT and become tumorigenic.

### ABL-mediated regulation of cytoskeletal dynamics and cell migration

Progression from an epithelial to a mesenchymal-like state is characterized by loss of cell–cell junctions, changes in cell polarity, and reorganization of the acto-myosin cytoskeleton to generate contractile forces to promote cell migration and directed cell movement [59]. Membrane protrusions, such as lamellipodia and filopodia, form at the leading edge of cells to promote cell migration [60]. ABL tyrosine kinases become activated during this transition and induce remodeling of the acto-myosin cytoskeleton [43, 61, 62]. ABL1 and ABL2 are 45% homologous within their F-actin binding C-terminal domains that allows both kinases to bind directly to the actin cytoskeleton [42, 43]. ABL2 has a second F-actin binding domain located in the internal [I/L]WEQ domain that allows ABL2 to bundle F-actin [41]. ABL1 is also capable of bundling actin through its G-actin binding domain (Fig. 1a).

In particular, ABL2 accumulates at sites of lamellipodia formation and can remodel the cytoskeleton by physically crosslinking microtubules and F-actin bundles through its microtubule and F-actin binding domains to promote cell protrusions [41]. ABL2 can also bind directly to microtubules and control microtubule behavior by promoting and directing filament extension [63]. In cervical cancer cells, ABL1 impacts microtubule assembly by phosphorylating PLK1, an enzyme that phosphorylates kinetochores, promoting kinetochore binding to the plus end of microtubules causing cytokinesis and tumor growth [64]. Further, the ABL PxxP motifs, which bind SH3 domains, regulate the actin cytoskeleton and promote filopodium dynamics and cell spreading by modulating the activity of SH3-domain adaptor proteins such as Crk and Nck [65].

The ABL kinases, primarily ABL2, can modulate cytoskeletal filament stability and elongation by binding to cytoskeletal effectors such as cortactin and members of the WASP-family verprolin-homologous (WAVE) complex, which activate the Arp2/3 complex to stimulate formation of new actin branches and actin filament stabilization [13]. The effects of ABL2 on actin polymerization are also mediated in part by targeting cortactin, an actin regulatory factor [66]. The internal (I/L)WEQ domain within ABL2 allows it to bind directly to actin where it cooperatively binds to the SH3 domain of cortactin via a Pro-rich motif in the ABL2 C-terminus [53, 66]. Together, ABL2 and cortactin stabilize actin filaments and promote actin nucleation through increased branching and severing as well as promote adhesion-dependent cell edge protrusions in fibroblasts [53, 66].

ABL2 can also regulate actin dynamics through its ability to bind directly and indirectly to the Wiskott-Aldrich syndrome protein (WASp) and WAVE family proteins. Both the WASP and WAVE family proteins contain a C-terminal verprolin homology/connecting region/acidic region (VCA) domain which mediates binding to actin monomers and activates the Arp2/3 complex, which is critical for the formation of actin-based membrane protrusions needed for cell migration and invasion during EMT [67–69]. The WASp and WAVE family proteins receive upstream signals from Rho-family small GTPases to enable VCA domain-mediated triggering of Arp2/3 actin polymerizing activity. Members of the WASp family including the WASp ortholog, N-WASp, contain an N-terminal Cdc42/Rac binding domain, which mediates interactions with the Rho GTPases. The WAVE isoforms (WAVE1, WAVE2, WAVE3) are regulated by indirect binding of Rac or Cdc42 [70–72]. ABL kinases can induce actin cytoskeleton remodeling in part by activating Rac1. It was shown that upon RTK stimulation, ABL1 and ABL2 tyrosine phosphorylate Sos-1, a guanine nucleotide-exchange factor (GEF), leading to Sos-1 mediated Rac1 activation and downstream signaling [73].

WAVE1 and -2 interact with the Abelson- (Abl) interactor (Abi) adaptor proteins, Abi-1 and -2, in the WAVE-regulatory complex (WRC), that is also comprised of the Nck-associated protein 125, p53-inducible PIR121 and, HSPC300. The WAVE2 complex was reported to regulate ABL activity, and in turn active ABL kinases can phosphorylate WAVE2 [74]. ABL1 phosphorylates WAVE2 on Tyr150 (Tyr151 in WAVE1 and WAVE3) [75, 76]. Analysis of the crystal structure of the WRC revealed that phosphorylation of Tyr150 is predicted to expose the VCA region allowing for interaction with the Arp2/3 complex [77]. Introduction of WAVE1 Y151E phospho-mimetic displayed high actin assembly activity while an un-phosphorylatable WAVE Y150F could not rescue actin polymerization [76, 77]. WAVE proteins were shown to become hyperphosphorylated in response to PDGF [78]. WAVE3 becomes tyrosine phosphorylated by ABL1 in response to PDGF causing stimulation of lamellipodia formation and cell migration [79]. These effects are inhibited by treatment with the ABL kinase ATP-binding site inhibitor STI-571 [79].

ABL kinases promote phosphorylation of WAVE proteins through interactions mediated by Abi proteins. Wave-1 was shown to bind to Abi-1 through a region within the Wave Homology Domain (WHD) [80]. Binding of Abi-1 to WAVE1 enhances WAVE complex formation, and both Abi-1 and WAVE1 are recruited to the tips of lamellipodia and filopodia to regulate actomyosin contractility during migration [80, 81]. Abi-1 also mediates coupling of ABL1 to WAVE2 promoting ABL1 tyrosine phosphorylation of WAVE2 to initiate actin polymerization and membrane remodeling at the cell periphery [75, 76, 82]. Initial reports suggested that Abi1 was released from the WAVE inhibitory complex to allow WAVE activation, but later findings showed that phosphorylation of WAVE2 enhances its association with Abi1 [75, 76, 82, 83]. This suggests a positive-feedback loop that allows sustained WAVE2 phosphorylation by ABL1. ABL1 can also phosphorylate WAVE3 in an Abi1-independent manner to stimulate lamellipodia formation and cell migration indicating additional modes of interaction [79].

ABL kinases also interact with WASP proteins and other downstream effectors to promote actomyosin contractility and cell migration. ABL kinases bind directly to N-WASp and release protein auto-inhibition [84, 85]. N-WASp exists in an inhibited state that can be relieved by either tyrosine phosphorylation or binding of the small GTPase Cdc42 [86–88]. Upon activation, the VCA domain is no longer occluded and is capable of binding to monomeric actin and the Arp2/3 complex to promote actin polymerization [84, 86, 89]. N-WASp binds directly to the SH3 domain of ABL2 allowing for ABL2 to phosphorylate Y256 on N-WASp [85]. Binding of the ABL2 SH3 domain and phosphorylation at Y256 increases N-WASp-mediated actin polymerization and increases localization of ABL and N-WASp to adhesion-dependent cell edge protrusions [85]. ABL kinases can also induce RhoA-dependent actomyosin contractility downstream of HGF/MET signaling to promote migration and invasion in breast cancer cells [90]. Further the ABL kinases have been implicated in migration of glioblastoma, melanoma, prostate, cervical, and hepatocellular carcinoma cells [19, 22, 91–95]. Thus, ABL kinases promote cell migration by targeting multiple pathways.

### ABL-mediated regulation of invadopodia and cancer cell invasion

Invadopodia are actin polymerization–driven protrusions that degrade the extracellular matrix (ECM) and facilitate cell invasion [96, 97]. Phosphorylation of cortactin serves as a master switch in invasive carcinoma cells during invadopodium formation and maturation [98]. Cortactin was initially identified as a substrate of the Src tyrosine kinase, but subsequent studies showed that the ABL kinases have a higher affinity for cortactin phosphorylation [53, 99]. The internal (I/L)WEQ domain of ABL2 binds directly to actin and is followed by a proline-rich motif in the ABL2 C-terminus that induces cooperative binding to the SH3 domain of cortactin [53, 66]. ABL2 binds to cortactin with greater affinity than ABL1 due to the substitution of arginine 161 and serine 187 in ABL1, to leucine 207 and threonine 233 in ABL2, respectively [100]. This interaction promotes ABL2-mediated phosphorylation of cortactin. Cortactin tyrosine phosphorylation is important for invadopodia formation as it promotes cortactin-mediated stabilization of N-WASp and cofilin allowing for generation of free actin barbed ends at invadopodia and increased invadopodia stability [101, 102]. ABL kinase phosphorylation of cortactin releases cortactin’s inhibitory interaction with cofilin allowing for cofilin to sever actin filaments, thus generating barbed ends for Arp2/3-dependent actin polymerization [53, 66]. ABL2 was shown to phosphorylate Y421 on cortactin following β1 integrin-ABL2 signaling allowing for an increase in cofilin-dependent barbed-ends required for formation of mature, degradation-competent invadopodia [103]. In the triple-negative breast cancer MDA-MB-231 cell line, stimulation of the epidermal growth factor receptor (EGFR) increased ABL2 activity and promoted cortactin-mediated invadopodia formation [104].

Sustained ABL kinase activity promotes maturation of functional invadopodia in breast cancer cells leading to increased invasion following stimulation of the chemokine receptor CXCR4 [105]. Upon CXCR4 stimulation by ligand, active ABL2 formed a complex with the membrane type-1 matrix metalloproteinase (MT1-MMP or MMP14), that localizes to invadopodia and promotes degradation of the ECM [105]. Similarly, ABL kinases drive melanoma cell invasion by inducing expression of matrix metalloproteinases *MMP-1*, *MMP-3*, and *MT1-MMP* [19]. ABL1 promotes melanoma invasion through STAT3-dependent *MMP1* expression, while ABL2 promotes melanoma invasion by increased expression of *MMP-1*, *MMP-3*, and *MT1-MMP* independently of STAT3.

In highly invasive breast cancer cells, ABL kinases were found to be constitutively activated downstream of deregulated ErbB receptors and Src kinases and promoted cancer cell invasion [106]. Treatment with the ABL ATP-site inhibitors imatinib or nilotinib decreased the invasive properties of some breast cancer cells. However, the effects of these inhibitors were cell context dependent [106, 107]. In a mouse xenograft model using MDA-MB-231 breast cancer cells, ABL2 knockdown resulted in larger primary tumor size, but decreased invasion, intravasation, and spontaneous metastasis to the lungs [2]. Further, using the same xenograft model, it was shown that the ABL ATP-site kinase inhibitors, imatinib or nilotinib, and the ABL allosteric inhibitor GNF-5, reduced invadopodia-mediated breast cancer cell metastasis by decreasing matrix metalloproteinase activity, cell invasion, and subsequent metastasis to the lungs [9].

## ABL kinases in solid tumor progression

Large-scale sequencing projects have identified increased expression of the ABL kinases in different solid tumor types due to *ABL* amplification, somatic mutations, and/or increased mRNA expression (reviewed in [12]). These findings are consistent with clinical reports analyzing patient samples for genomic and/or gene expression changes in high-grade pancreatic, renal, colorectal, breast and gastric tumors [108–111]. Studies examining Kaplan–Meier survival curves of lung, breast, colorectal, hepatocellular carcinoma patients of varying subtypes found that elevated ABL1 and/or ABL2 is associated with decreased metastasis-free survival and/or lower overall survival [4, 6, 11, 94, 112].

**Fig. 2:**
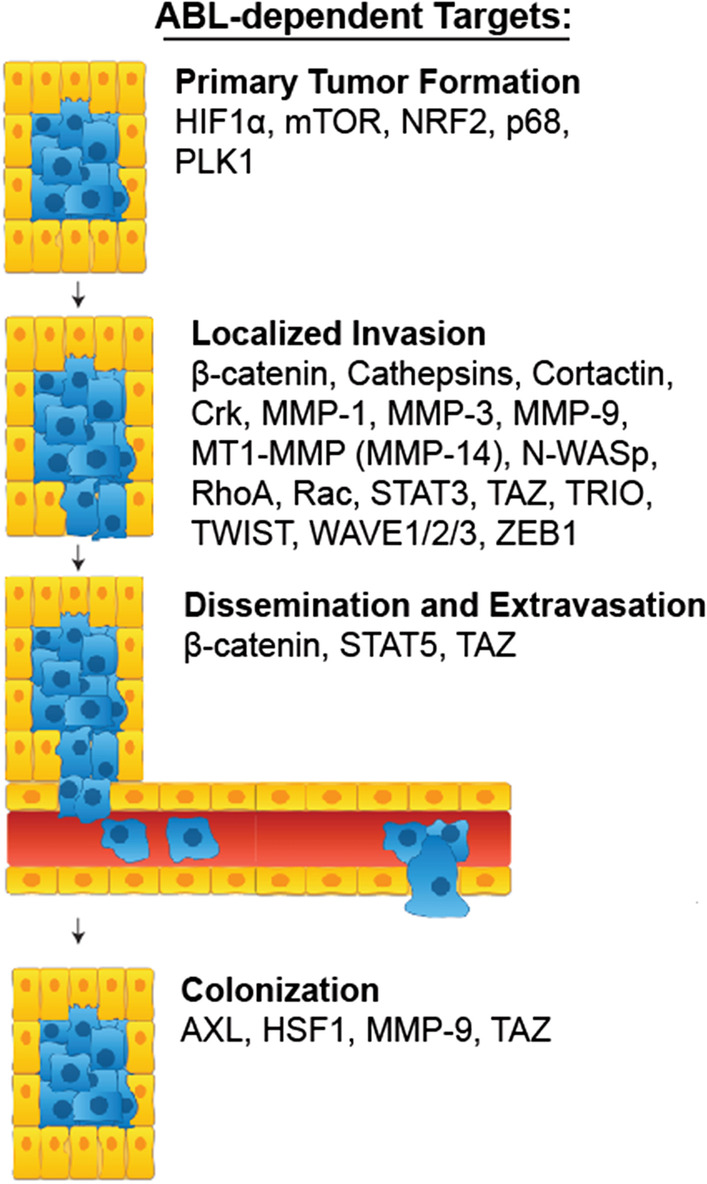
ABL-dependent targets promote EMT and metastasis. Activated ABL kinases and their downstream signaling targets promote tumor progression and metastasis by targeting distinct processes required for tumor growth, invasion, dissemination, extravasation and colonization of distal sites

The consequences of elevated expression of ABL1 and ABL2 for tumor progression are cell context-dependent. Single knockdown of either ABL1 or ABL2 in triple-negative breast cancer cells impaired anchorage independent growth, while expression of a constitutively active form of ABL1 in 4TI murine mammary tumors inhibited tumor growth [29, 106]. Consistent with these findings, knockdown of ABL2 in triple-negative MDA-MB-231 breast cancer xenografts promoted tumor growth via increased cell proliferation [2]. In contrast, depletion of ABL1 and ABL2 in MCF7 cells impairs the growth of MCF7 xenograft tumors [113]. While the effects of ABL1 or ABL2 inhibition has mixed effects on primary breast tumor growth, genetic or pharmacologic inhibition of the kinases impairs breast cancer metastasis [2, 9, 11]. Notably, ABL1 plays a critical role in an aggressive form of hereditary kidney cancer observed in patients with a germline mutation in the enzyme fumarate hydratase (FH) that leads to the development of hereditary leimyomatosis and renal cell carcinoma (HLRCC) [114]. In these cells, ABL1 signals through mTOR and HIF1α to upregulate aerobic glycolysis and neutralize proteotoxic stress by promoting nuclear accumulation of NRF2, a transcription factor that activates a cell detoxification program (Fig. 2). Inactivation of ABL1 markedly inhibited the growth of HLRCC xenografts [114].

Preclinical mouse models have shown that genetic or pharmacologic inhibition of the kinases has a greater impact impairing cancer cell metastasis of solid tumors in comparison to primary tumor growth [2, 4–6, 9, 17–19]. The predominant effects of the ABL kinases on metastatic phenotypes might be due to cumulative regulation of the epithelial to mesenchymal transition and other distinct steps of the metastatic cascade required for initiation, dissemination and colonization of distal sites [115–118].

### ABL kinases promote metastasis

Several reports have shown a requirement for ABL kinases in breast cancer metastasis using preclinical mouse models. Knockdown of ABL2 in breast cancer cells resulted in decreased spontaneous metastasis to the lungs following orthotopic implantation of breast tumors in the mammary fat pad [2]. Using an intracardiac mouse model of metastasis, shRNA-mediated knockdown of both ABL1 and ABL2 in bone tropic triple-negative breast cancer cells decreased metastasis to the bone and increased overall survival [11]. Importantly, this study demonstrated that ABL-dependent activation of the TAZ and STAT5 transcription factors was required for breast cancer metastasis to the bone (Fig. 2). Further, treatment with ABL kinase pharmacologic inhibitors reduced breast cancer spontaneous metastasis to the lungs [9].

Following intracardiac injection of non-small cell lung cancer (NSCLC) cells in preclinical mouse models, it was found that the ABL kinases, specifically ABL2, drove metastasis of lung cancer cells to distal sites in the body including the brain [4–6]. Genetic and pharmacologic inhibition suppressed metastasis and increased overall survival in mice. Global transcriptome analysis revealed that the ABL kinases are required for expression of pro-metastasis genes [4]. Specifically, ABL kinases promote stability of the transcriptional coactivators TAZ and β-catenin by decreasing their interaction with the β-TrCP ubiquitin ligase [4]. Both TAZ and β-catenin have been implicated in EMT. Active TAZ can promote tumorigenesis by increasing cell proliferation, metastatic colonization, chemoresistance, and EMT [28, 119, 120]. In this regard, β-catenin is expressed at the invasive front of colorectal carcinomas and upregulates a pro-invasive gene expression profile during colorectal metastasis [121, 122]. Interestingly, it was found that ABL2 and TAZ activate an autocrine signaling loop during lung adenocarcinoma metastasis to promote colonization of the brain parenchyma [6]. The ABL2–TAZ signaling axis induces expression of multiple targets including the AXL RTK that engages with ABL2 protein kinase in bi-directional signaling (Fig. 2). ABL2 targets distinct transcriptional regulatory networks that include the heat shock factor 1 (HSF1) in lung adenocarcinoma cells to drive brain colonization through increased lung cancer cell survival and outgrowth (Fig. 2) [5]. Further, lung cancer cells harboring shRNAs against the ABL kinases exhibited decreased extravasation from blood vessels into lung tissue in preclinical mouse models of metastasis [4]. Recently, ABL kinases were shown to be activated by co-culture of lung adenocarcinoma cells with mesenchymal stem cells (MSCs) leading to ABL-mediated MMP-9 expression, secretion, activation of MMP-9 proteolytic activity and metastasis [17] (Fig. 2). Both ABL1 and ABL2 kinases are required for metastasis of MSC-primed lung cancer cells to distal sites following intracardiac injection in mouse models.

ABL kinases target diverse protein signaling pathways to facilitate EMT and metastasis. In hepatocellular carcinomas, ABL1 was shown to be important for claudin1 expression [10, 22]. Claudin1 promotes a malignant phenotype by inducing expression of the EMT transcription factors Slug and Zeb1 leading to repression of cell adhesion proteins and increased cell motility [10]. ABL1 and ABL2 promote invasion and metastatic progression of melanomas in part by activating the transcription factors Ets1, Sp1, and NF-κB/p65 which induce expression of cathepsin ECM proteases [19, 21]. In colorectal cancer, when the tumor suppressor gene *Aes* is knocked out NOTCH1 becomes activated and stimulates ABL1 activity [112]. ABL1 then phosphorylates the Rac/RhoGef protein TRIO on Y2681 causing Rho activation and colorectal cancer cell invasion. Phosphorylation of Y2681 on TRIO is correlated with poor colorectal cancer patient prognosis and inhibition of ABL suppressed cancer cell invasion in mice [112]. In non-*Aes* mutant colorectal cancer, ABL1 can also increase NOTCH1 and MYC protein levels leading to enhanced tumor growth [123]. Additionally, ABL1 can become activated following PDGF stimulation leading to phosphorylation of Y593 on the nuclear RNA helicase p68. Phosphor-p68 then promotes nuclear translocation of β-catenin and stimulation of EMT [47]. These findings highlight the ability of the ABL kinases to promote metastasis by modulating a vast array of substrates in diverse signaling pathways necessary for cellular processes that contribute to EMT and subsequent steps in the metastasis cascade (highlighted in Fig. 2).

### ABL kinases promote chemoresistance

Recent studies have demonstrated that that transition into a mesenchymal state promotes resistance to chemotherapy [124, 125]. Some of the molecular signaling pathways employed by cells undergoing EMT are similar to the ones utilized by cancer cells to acquire a therapy resistant, stem-like state to escape conventional therapies. Interestingly, preclinical studies of mice harboring therapy resistant *Kras*^*G12D/*+^; *p53*^*−/−*^ lung tumors treated with an ABL kinase inhibitor sensitized these tumors to standard of care docetaxel chemotherapy [18]. Tumors treated with vehicle control existed in a dedifferentiated, mesenchymal state, and upon treatment with the ABL allosteric inhibitor GNF-5, the lung cancer cells underwent differentiation into an epithelial-like state making the tumors more susceptible to docetaxel. Further, profiling of *BRAF* mutant melanomas with acquired resistance to BRAF and MEK inhibitors found that ABL kinase activity was increased in therapy resistant cells [126]. Following treatment with nilotinib or genetic inhibition of ABL1/ABL2, cancer cells were sensitized to BRAF and MEK inhibitors and underwent cell death as well as decreased tumor growth. These studies suggest that the ABL kinases likely play a role in therapy resistance. However, additional studies are needed to understand the diverse ABL-regulated mechanisms implicated in therapy resistance in distinct tumor types.

## Therapeutics targeting ABL kinases

Among the clinically available tyrosine kinase inhibitors (TKIs), some of the most successful to date target the ABL kinases, specifically in the context of BCR-ABL driven chronic myelogenous leukemia (CML) (Table 1). ABL kinases are also activated in many solid tumors, expanding the utility of ABL kinase inhibitors, and particularly the highly specific ABL allosteric inhibitors have shown efficacy in the treatment of metastatic lung cancer [4, 5]. Importantly, the available ATP-competitive ABL kinase TKIs target multiple tyrosine kinases in addition to ABL1 and ABL2 whereas the ABL allosteric inhibitors are specific to the ABL kinases (Table 1). The ability of the ATP-competitive inhibitors to target other kinases, such as PDGFRA and KIT, has made these inhibitors efficacious in the treatment of selective solid tumors that require the activity of such kinases to promote tumor growth. However, in tumors where these mutations are not driving growth, the necessary concentration needed to effectively inhibit disease progression by non-selective ATP-competitive inhibitors may result in detrimental off-target effects due to the promiscuous nature of the ATP-site inhibitors. Therefore, using ABL inhibitors that are highly specific and mono-selective, such as the allosteric inhibitors and PROTACS, might be a more effective strategy for developing future therapies.

**Table 1:** ABL kinase inhibitors

Name	Alternative name	Inhibitor type	Targets	References describing inhibitor targets	Clinical trials in solid tumors	Regulatory status
Imatinib	Gleevec/STI57	ATP-site, Type II	ABL1, ABL2, BCR-ABL1, CSF1R, DDR1, DDR2, KIT, NQO2, PDGFR1	[160]	**Yes** **Non-small cell lung cancer:** NCT01011075; NCT00408460 **Breast Cancer:** NCT00193180; NCT00338728; NCT00087152 **Gastrointestinal tumors:** NCT01151852; NCT00867113 **Melanoma:** NCT00424515; NCT00470470	FDA approved for CML, Ph + ALL, MDS/MPD, ASM, HES/CEL, DFSP, GIST
Dasatinib	Sprycel/ BMS-354825	ATP-competitive, Type I	ABL1, ABL2, BCR-ABL1, BLK, BTK, CSK, CSR1R, DDR1, DDR2, EGFR, ERBB2, FGR, FRK, FYN, GAK, GCK, HCK, ILK, KIT, LCK, LIMK1, LIMK2, LYN, MAP2K, MAP3K, MAP4K, PDGFR, RIPK2, SLK, SRC, SYK, TEC, TYK2, YES1,	[160–162]	**Yes** **Non-small cell lung cancer:** NCT00858403; NCT00826449; NCT01999985 **Breast Cancer:** NCT00817531,NCT00924352, NCT00767520, NCT00410813 **Gastrointestinal tumors:** NCT00568750; NCT01643278;NCT00504153 **Melanoma:** NCT00597038;NCT00436605	FDA approved for CML, Ph + ALL
Nilotinib	Tasigna/AMN107	ATP-site, Type II	ABL1, ABL2, BCR-ABL1, CSF1R, DDR1, DDR2, KIT, NQO2, PDGFR	[160]	**Yes** **Breast Cancer:** NCT04205903 **Gastrointestinal tumors:** NCT00976612; NCT00471328 **Melanoma:** NCT01395121; NCT00788775; NCT01099514	FDA approved for CML
Bosutinib	Bosulif/SKI-606	ATP-competitive, Type I	ABL1, ABL2, BCR-ABL1, CAMK2G, CDK2, HCK, LYN, MAPKK1, MAPKK2, MAPKKK2, SRC	[163–165]	**Yes** **Non-small cell lung cancer:** NCT03023319 **Breast Cancer:** NCT00793546; NCT00319254; NCT00880009; NCT03854903; NCT00959946; NCT00759837 **Advanced Solid Tumors:** NCT03297606; NCT01001936	FDA approved for CML, Ph + CML
Ponatinib	Iclusing/AP24534	ATP-site, Type II	ABL1, ABL2, BCR-ABL1, BLK, CSFR1, DDR1, DDR2, EPHRs, FGFR1, FGFR2, FGR, FLT3, FRK, FYN, HCK, LCK, LYN, RET, SRC, TEK, TIE2, TRKA, TRKB, TRKC, PDGFR, VEGFR1, VEGFR2, VEGFR3, YES1	[166–169]	**Yes** **Non-small cell lung cancer:** NCT01761747; NCT03704688; NCT01935336; NCT01813734; NCT01813734; **Breast Cancer:** NCT03878524; NCT04591431 **Gastrointestinal tumors:** NCT01874665	FDA approved for CML, Ph + ALL
Axitinib	Inlyta/AG013736	ATP-competitive, Type I	BCR-ABL1 (T315I), KIT, PDGFR, VEGFR1, VEGFR2, VEGFR3	[170]	**Yes** **Non-small cell lung cancer:** NCT03472560; NCT00094094; NCT00094094 **Breast Cancer:** NCT00076024; **Gastrointestinal tumors:** NCT00700258 **Renal Cell Carcinoma:** NCT02493751; NCT00678392; NCT00920816; NCT02579811	FDA approved for Renal Cell Carcinoma
Vandetanib	Caprelsa/ZD-6474	ATP-competitive, Type I	ABL1, EGFR, RET, VEGFR	[171]	**Yes** **Non-small cell lung cancer:** NCT01586624; NCT00753714; NCT01823068 **Breast Cancer:** NCT01934335; NCT00481845; NCT00494481 **Gastrointestinal tumors:** NCT02015065 **Thyroid Cancer:** NCT01876784; NCT01496313;	FDA approved for Advanced Medullary Thyroid Cancer
GNF2, GNF5		Allosteric	ABL1, ABL2, BCR-ABL1	[147]	**No**	Not FDA approved
ABL001	Asciminib	Allosteric	ABL1, ABL2, BCR-ABL1	[148]	**Yes-** **Advanced solid tumors:** NCT04492033; NCT03292783	Phase III Clinical Trials in CML
DAS‐6‐2‐2‐6‐CRBN		PROTAC	BCR-ABL1	[155]	**No**	Preclinical Studies Only
BOS‐6‐2‐2–6‐CRBN		PROTAC	BCR-ABL1	[155]	**No**	Preclinical Studies Only
GMB-475		PROTAC	BCR-ABL1	[154]	**No**	Preclinical Studies only

### ATP-competitive ABL inhibitors

Classical ABL tyrosine kinase inhibitors can be stratified into classes based on their mechanism of action. ATP competitive inhibitors target the ATP binding pocket of the kinase domain and can be further subdivided into type 1 or type 2 based on whether they target the active or inactive conformation of the kinase domain. Imatinib (Gleevec) was the first TKI developed against BCR-ABL. It binds the ATP-binding site of ABL1 and inhibits both BCR-ABL1 and ABL1 resulting in inhibition of cell proliferation and apoptosis of leukemic cells [127–130]. Treatment of early chronic phase CML patients with Imatinib as a first line therapy leads to durable remission and a stark improvement in 5 year overall and progression free survival [131]. However, the relapse rate among patients with advanced or blast crisis phase CML is high due to the development of drug resistance mutations in the ABL kinase domain. This clinical need led to the development of several second and third generation TKIs targeting BCR-ABL including: Dasatinib, Nilotinib, Bosutinib, and Ponatinib [132] (Table 1). Dasatinib and Nilotinib have been FDA approved as first and second line therapy, and Ponatinib and Bosutinib have been approved as second line therapy for Ph + leukemia patients with BCR-ABL mutations [132]. Additionally, Axitinib, a vascular endothelial growth factor receptor (VEGFR) inhibitor has been shown to inhibit the drug resistant gate keeper mutant of BCR-ABL [133]. Vandetanib, originally designed as a VEGFR2 inhibitor, has also been shown to be a potent inhibitor of a number of kinases including ABL and has been FDA approved for the treatment of medullary thyroid carcinoma (Table 1) [134, 135]. Type 1 ATP competitive inhibitors including Dasatinib, Bosutinib, Vandetanib, and Axitinib target the active conformation of the kinase domain. Conversely, type 2 ATP competitive inhibitors including Imatinib and Nilotinib target the inactive conformation of the kinase domain [136, 137] (Table 1).

Clinical trials have effectively used the ATP-site inhibitors imatinib and nilotinib to treat melanoma patients harboring c-Kit mutations as these drugs can target the c-Kit receptor kinase in addition to ABL and other tyrosine kinases [138–140]. However, clinical trials designed to use the ATP-site inhibitors in non-c-Kit mutant solid tumors because of their ability to target multiple tyrosine kinases, such as PDGFR, Kit, DDR1/2, or Src, were ineffective [136, 137, 141–145]. The lack of efficacy could be due in part to toxicity elicited by effective tumor killing doses or activation of alternative cell survival pathways [136, 137, 145]. These findings have been further substantiated by preclinical data showing that treatment with imatinib induces activation of the RAF-ERK pathway in cancer cells [4, 11, 146].

### ABL allosteric inhibitors

Allosteric ABL inhibitors bind to regulatory regions that inhibit kinase activity. Unlike ATP competitive inhibitors, allosteric inhibitors are highly specific for ABL kinases and effectively target ABL1, ABL2, as well as the BCR-ABL1 fusion protein. The first allosteric ABL inhibitor to be described was GNF2, a compound that bound to the myristate binding cleft of ABL [147]. To circumvent inherently limiting pharmacokinetic properties of GNF-2, GNF-5 a structural analog of GNF-2 was designed and shown to have similar inhibitory properties to GNF-2 [147]. Treatment with GNF-5 effectively decreased tumor burden in mice harboring BCR-ABL1 leukemias and sensitized ATP-site inhibitor resistant leukemias to the ATP competitive TKIs [147]. Recently, the ABL allosteric inhibitor ABL001 (Asciminib) which binds to the myristoyl binding site with a higher affinity than GNF-2/5 [148], has been evaluated in multicenter clinical trials in patients with CML and Ph + ALL (NCT02081378, NCT03292783) (Table 1). More recently, Asciminib was used in a Multicenter Phase 3 Study in CML chronic phase patients that had been previously treated with two tyrosine kinases [149]. Notably, ABL allosteric inhibitors have also been shown to be efficacious in preclinical mouse models of breast and lung cancer metastasis, as treatment with GNF5 or ABL001 decreased lung adenocarcinoma metastasis to the brain and breast cancer metastasis to the bone [4, 6, 11]. Unexpectedly, recent work uncovered functional differences between ABL allosteric versus ATP-competitive inhibitors as the pro-metastatic ABL2-HSF1 complex was completely disrupted by GNF5 treatment, but was largely unaltered after treatment with the ATP-competitive inhibitor Nilotinib [5]. These exciting findings support the notion that allosteric and ATP-competitive inhibitors have differential effects on the protein-interactome of the ABL kinases, which suggests that these drugs could have distinct therapeutic effects in cancer cells and patients harboring solid tumors.

### Emerging strategies to target ABL kinases: PROTACs

While established ABL inhibitors have been instrumental in the treatment of CML and have emerging potentials in solid tumors, treatment with these inhibitors can result in drug resistance. Resistance mechanisms could be due to previously described mutational changes as well as residual scaffolding functions of ABL outside of its kinase activity [150, 151]. Proteolysis Targeting Chimera (PROTAC) technology is an emerging therapeutic strategy that could be useful to impair ABL expression and function. PROTACs are bifunctional small molecules designed to target both the target protein as well as an E3 ubiquitin ligase to induce degradation of the target protein [152]. Recent studies designing PROTACs that effectively degrade ABL1, ABL2 and BCR-ABL1 have been successful in vitro [153–155]. ABL was partially degraded by targeting the kinase domain using either Bosutinib and a ligand that recruits E3 ligase Cereblon, or -Dasatinib fused to ligands for either Cereblon or VHL [155]. However, because both Bosutinib and Dasatinib target multiple protein kinases other than ABL (Table 1), it is likely that the effects of these PROTACS are mediated by degradation of several kinases. A recent study showed enhanced sensitivity and complete degradation of ABL by targeting the myristoyl binding pocket of ABL and VHL-mediated degradation [154]. While these early studies have only been conducted in vitro, they are promising for potential future in vivo preclinical and clinical studies.

### Effect of ABL Inhibitors on EMT phenotypes

Much like the contribution of ABL to EMT, ABL inhibitors have been demonstrated to have context dependent effects on EMT phenotypes. Treatment of mesenchymal- like triple negative breast cancer cells with Dasatinib led to a decrease in cell invasion but not migration, while treatment of “normal” mammary epithelial cells with Imatinib induced an EMT phenotype through loss of cell–cell junctions [29, 156]. Another study reported that breast cancer cells treated with Imatinib had suppressed EMT [157]. Treatment of prostate cancer cells with Dasatinib induced a more epithelial phenotype and increased expression of E-Cadherin [158]. Treatment of melanoma cell lines with Nilotinib or GNF-2 led to a context dependent decrease in cathepsin, and invasion [21]. Bosutinib has been shown to inhibit the migration and invasion of KRAS mutant non-small cell lung cancer cells [159]. Together, these data suggest that inhibition of ABL may be an effective approach to combat EMT but only in certain cellular contexts.

## Conclusions

ABL kinases function as a signaling nexus regulating cellular processes critical for the epithelial–mesenchymal transition and multiple subsequent steps in the metastatic cascade. ABL kinases are capable of promoting distinct processes required for EMT, but their role is cell context dependent. In non-transformed epithelial cells, the ABL kinases maintain cell–cell and cell–matrix contacts but in transformed cells they promote EMT and disease progression. These disparate effects may be dependent on the levels of ABL kinase activity, which are markedly elevated in metastatic tumors compared to non-transformed cells. Further, ABL kinases are capable of binding directly and indirectly to the actomyosin cytoskeleton to promote motile and invasive forces. Accumulating evidence supports the potential use of ABL kinase inhibitors to impair solid tumor progression and metastasis. Preclinical studies revealed that the ABL kinases promote cancer cell invasion, dissemination, extravasation, and colonization. While clinical trials using the ABL ATP-site inhibitors, which target multiple substrates, have failed to extend distant metastasis-free survival of patients with certain solid tumors, such as breast and lung, recent preclinical studies using the highly specific ABL allosteric inhibitors, GNF5 and ABL001 (Asciminib) are promising. Future studies are needed to dissect the roles of ABL kinases in solid tumor progression and metastasis, and to understand how the ABL kinases facilitate chemoresistance. Exciting new data is emerging on the efficacy of incorporating ABL inhibitors into current standard of care treatment regimens that could benefit patient response and overall survival.

## Data Availability

Not applicable.

## Abbreviations

Abi: Abelson-interactor protein
ABL1: Abelson tyrosine kinase; cellular homolog of the transforming factor co-opted by the Abelson virus (c-ABL)
ABL2: Abelson proto-oncogene 2; or ABL-related gene (ARG)
AXL: AXL receptor tyrosine kinase
BCR-ABL1: Breakpoint cluster region-Abelson kinase
BRAF: B-raf proto-oncogene
B-TrCP: Beta-transducin repeat containing E3 ubiquitin ligase
Cdc42: Cell division cycle 42
CML: Chronic myelogenous leukemia
Crk: Adapter molecule Crk
CXCR4: C-X-C motif chemokine receptor 4
DDR1/2: Epithelial discoidin domain-containing receptor 1 or 2
ECM: Extracellular matrix
EGFR: Epidermal growth factor receptor
EMT: Epithelial to mesenchymal transition
ErbB: Epidermal growth factor receptor 2
Ets1: ETS proto-oncogene 1
FH: Fumarate hydratase
GEF: Guanine nucleotide-exchange factor
HIF1α: Hypoxia inducible factor 1 subunit alpha
HGF: Hepatocyte growth factor
HLRCC: Hereditary leimyomatosis and renal cell carcinoma
HSF1: Heat shock factor 1
KIT: KIT Proto-Oncogene, Receptor Tyrosine Kinase
MEK: Mitogen-activated protein kinase kinase 1
MET: MET receptor tyrosine kinase
MMP: Matrix metalloproteinase
MSC: Mesenchymal stem cell
mTOR: Mechanistic target of rapamycin kinase
Nck: Non-catalytic region of tyrosine kinase adaptor protein 1
NES: Nuclear export sequence
NLS: Nuclear localization sequence
NOTCH: Notch homolog 1
NRF2: Nuclear factor erythroid 2-related factor 2
nRTK: Non-receptor tyrosine kinase
p65: Nuclear factor NF-kappa-B p65 subunit
p68: DEAD box protein 5 RNA helicase
PDGF: Platelet-derived growth factor
Rac1: Ras-related C3 botulinum toxin substrate 1
Rap1: Ras-related protein 1
Rho: Rhodopsin
RTK: Receptor tyrosine kinase
SH: Src homology
shRNA: Short hairpin RNA
Slug: Snail family transcriptional repressor 2
SNAIL: Snail family transcriptional repressor 1
sp1: Specificity protein 1
Src: Schmidt-Ruppin A-2 tyrosine kinase
STAT3: Signal transducer and activator of transcription 3
STAT5: Signal transducer and activator of transcription 5
TAZ: Tafazzin
TKI: Tyrosine kinase inhibitor
TRIO: Trio Rho guanine nucleotide exchange factor
TWIST: Twist family BHLH transcription factor 1
VCA: Verprolin homology/connecting region/acidic region
VHL: Von Hippel-Lindau
WASp: Wiskott-Aldrich syndrome protein
WAVE: WASP-family verprolin-homologous
WRC: WAVE-regulatory complex
WHD: Wave Homology Domain
Y: Tyrosine
ZEB1: Zinc finger E-Box binding homeobox 1
